# Multiprofessional Treatment of High Blood Pressure in Very Elderly Patients

**DOI:** 10.5935/abc.20160196

**Published:** 2017-01

**Authors:** Luciana Muniz Sanches Siqueira Veiga Jardim, Thiago Veiga Jardim, Weimar Kunz Sebba Barroso de Souza, Camila Dutra Pimenta, Ana Luiza Lima Sousa, Paulo César Brandão Veiga Jardim

**Affiliations:** Arterial Hypertension League of the Federal University of Goiás, Goiânia, GO - Brazil

**Keywords:** Hypertension, Aged, 80 and over, Patient Care. Team, Aging, Cohort Studies

## Abstract

**Background:**

As the world population ages, patients older than 80 years, known as very elderly, are more frequently found. There are no studies in this age group aimed at analyzing the multidisciplinary intervention in the treatment of systemic arterial hypertension (SAH) and some comorbidities.

**Objectives:**

To assess the effect of a multidisciplinary approach in very elderly hypertensives cared for at a specialized service.

**Methods:**

Longitudinal retrospective cohort study in a multidisciplinary service specialized in the SAH treatment in the Brazilian West-Central region. Patients aged 80 years and older by June 2015 were included. Data from the first (V1) and last visit (Vf) were assessed. Anthropometric variables, blood pressure (BP), renal function, pharmacological treatment, lifestyle, comorbidities and cardiovascular events were studied, comparing data from V1 and Vf. Controlled BP was defined as systolic blood pressure (SBP) lower than 140 mm Hg and diastolic blood pressure (DBP) lower than 90 mm Hg. Statistical analyses were performed with SPSSR software, version 21.0. Values of p<0,05 were considered significant.

**Results:**

Data of 71 patients were assessed with a mean follow-up time of 15,22 years. Their mean age at V1 was 69.2 years, and, at Vf, 84.53 years, and 26.8% of them were males. There was a significant reduction in mean SBP (157.3 x 142.1 mm Hg; p<0.001) and DBP (95.1 x 77.8 mm Hg; p<0.001), with an increase in BP control rates from V1 to Vf (36.6 x 83.1%; p<0.001). The number of antihypertensive drugs used increased (1.49 x 2.85; p<0.001), with an increase in the use of angiotensin-converting enzyme inhibitors (22.5 x 46.5%; p=0.004), angiotensin II receptor blockers (4.2 x 35.2%; p<0.001) and calcium-channel blockers (18.3 x 67.6%; p<0.001). There was a reduction in total cholesterol (217.9 x 191 mg/dL; p<0.001) and LDL-cholesterol (139.6 x 119.0 mg/dL; p<0.001), but worsening of the glomerular filtration rate (62.5 x 45.4 mL/min; p<0.001).

**Conclusion:**

The multidisciplinary intervention in very elderly hypertensives increased BP control rate, with optimization of the pharmacological treatment.

## Introduction

From the chronological viewpoint, elderly are defined as individuals aged 65 years and older living in developed countries, or aged 60 years and older living in developing countries.^1^ In that age group, those who have reached the eighth decade are called 'oldest old' or 'very elderly'.^2^

Aging, regardless of ethnical, social and cultural factors inherent in each population, is associated with a higher probability of chronic noncommunicable diseases (NCDs) secondary to morphophysiological and functional changes, as well as to lifestyle.^3^

Systemic arterial hypertension (SAH) is the most common NCD among the elderly.^4^ Its prevalence increases progressively as age advances, SAH being considered the major modifiable risk factor for cardiovascular disease in the geriatric population.^5^ There is a direct and linear relationship of blood pressure (BP) and age, the prevalence of SAH being higher than 60% in those older than 65 years.^6^

Because SAH is a multifactorial clinical syndrome, a multiprofessional team to support hypertensives is desirable whenever possible.^7,8^ That team should comprise all professionals who manage hypertensives,^9,10^ an initiative recommended in national and international guidelines.^11,12^

To our knowledge, there is no study in very elderly hypertensives confirming the benefit of the multiprofessional management.

This study was aimed at assessing the result of the multiprofessional treatment of very elderly hypertensive patients undergoing regular follow-up in a reference service for the multidisciplinary treatment of SAH.

## Methods

This study was assessed and approved by the Ethics Committee in Human and Animal Medical Research of the institution (protocol 700.942 of 06/26/2014).

This study assessed retrospectively data of very elderly patients undergoing regular follow-up in a multiprofessional service of reference for the treatment of SAH in the Brazilian West-Central region.

The service has been existing for more than 20 years, dedicated to the multiprofessional care of hypertensive patients, teaching and research. Its professional team consists of physicians (cardiologists, endocrinologists and nephrologists), nurses, nutritionists, physical therapists, physical education teachers, psychologists and musical therapists. Patients are followed up at maximum 3-month intervals between the appointments, regardless of the health specialty responsible for the appointment. In addition, educational and health promotion activities are routinely performed.

The medical team assesses symptoms, lifestyle habits and medications used, performs complete physical examination, interprets the complementary tests and establishes the management, which includes: prescription of drugs and nonpharmacological measures; request of complementary tests; and scheduling of return appointments, with definition of the time interval and designation of the assisting professional. In addition, if clinical decompensation is identified in the medical consultation, the patient is referred to emergency care or hospitalization.

The nurse team assesses symptoms, vital signs, lifestyle habits and medications used, in addition to instructing about treatment adherence in both pharmacological and nonpharmacological aspects. They define the interval of the nurse return appointment and refer patients for medical consultation, when necessary or when the time since the last medical consultation is longer than 6 months.

The group of nutritionists emphasizes nonpharmacological aspects of care, specifically the diet. They collect dietary data and assess anthropometric data and vital signs. The management is aimed at dietary guidance with emphasis on salt restriction and prescription of special diets, when necessary.

The other health specialties of the service do not conduct formal appointments, but rather a series of educational interventions to promote health with the hypertensive patients. The physical therapists and physical education teachers conduct periodical meetings previously scheduled or meet with patients at the waiting room to emphasize the importance of regular physical activity practice and preventive measures of injuries and falls. In addition, they promote assisted group physical activity for patients. Similarly, the psychology and musical therapy teams act mainly in the waiting room, providing instructions and interventions aimed at stress reduction and waiting time improvement.

Since the beginning of the multidisciplinary service more than 20 years ago, consultations have been registered in a standardized form, whose completion by all health professionals is mandatory, ensuring data reliability and reproducibility throughout the follow-up years.

This study included patients aged 80 years and older by June 2015, with at least three consultations attended in the service and reported in the medical record. We collected data of the first consultation, with the patient already diagnosed with SAH and on conventional treatment (nonmultiprofessional) at another health service. Those data were compared with the data of the last consultation at our service reported in the medical record after the institution of multiprofessional treatment, regardless of the time elapsed between both.

The treatment goals established for the very elderly followed the recommendations of the national guidelines at the time, which establish the management adopted at our service since the beginning of its activities. That management abides by the updates and changes of those guidelines.

Controlled BP was defined as systolic blood pressure (SBP) < 140 mmHg and diastolic blood pressure (DBP) < 90 mmHg, in accordance with the recommendations of national guidelines.^11^

The following data were collected from the medical records:

**Anthropometric data:**

- Consisted in weight, height and calculation of body mass index (BMI) with the Quetelet formula (BMI = weight in kg/height^2^ in meter).

**Blood pressure:**

- The measures were taken with a mercury-column manometer after 5 minutes of rest, twice, at a 2-minute interval, on the upper limb, with the individual sitting with the arm supported. The mean of the last two measures was considered for data analysis.

**Laboratory data:**

- Renal function with creatinine measure;
- Creatinine clearance calculated with the MDRD formula;^13^
- Fasting glycemia and lipid panel: collected after a 12-hour fasting, and observing the recommendation of no alcoholic beverage consumption in the preceding 48 hours. The enzymatic colorimetric method was used to determine total cholesterol (TC), HDL-cholesterol (HDL), serum triglycerides (TG) and glycemia. The LDL-cholesterol (LDL) level was estimated with the Friedewald formula:^14^ LDL = TC - (HDL + TG/5).

**Medications being used:**

- Anti-hypertensive drugs: analyzing the number and classes of drugs;
- Other drugs: statins and acetylsalicylic acid.

**Lifestyle:**

- Smoking: smoker or nonsmoker;
- Alcoholism: alcoholic beverage consumption or not;
- Sedentary lifestyle:
  - sedentary - no leisure physical activity
  - non-sedentary - any type of leisure physical activity.

**Associated comorbidities:**

- Dyslipidemia and diabetes mellitus.

**Cardiovascular events:**

- Acute myocardial infarction (AMI) - AMI reported in the medical record and confirmed by hospital discharge summary and/or altered levels of tissue necrosis markers;
- Stroke - reported in the medical record and confirmed by hospital discharge summary and/or imaging exam suggestive of cerebrovascular event;
- Need for surgical myocardial revascularization or angioplasty - reported in the medical record and confirmed by hospital discharge summary, surgeon's report and/or angioplasty report.

### Data bank and statistical analysis

Data were stored in a data bank structured in Excel (Microsoft) and analyzed comparatively. Statistical analysis was performed using the SPSS software (*Statistical Package of Social Science*, version 21.0, Chicago, IL, USA). Kolmogorov-Smirnov test was used to check if the continuous variables had a normal distribution. Paired Student *t* test was used to compare the numerical variables, expressed as mean and standard deviation. Qualitative variables were compared using McNemar test. The significance level adopted was p<0.05.

## Results

This study assessed 71 very elderly patients on regular follow-up at our service. The mean follow-up time was 15.22 years (ranging from 3 months to 23.5 years), 85.9% of the patients were followed up for more than 5 years, and only two patients for less than 1 year.

Male patients accounted for 26.8% of the sample. The patients' mean age at the first visit was 69.2 years (range, 57 to 91 years), and, at the final visit, 84.53 years (range, 80 to 94 years).

The BP control rate, which was initially 36.6% (n=26) with conventional treatment, passed to 83.1% (n=59) (p<0.001).

Mean BP levels decreased significantly during follow-up, with an increment in the number of anti-hypertensive drugs used and optimization of the drug classes prescribed. That optimization was characterized by an increased use of the first-line drug classes [angiotensin-converting-enzyme inhibitors (ACEI), angiotensin receptor blockers (ARB) and calcium-channel blockers (CCB)] (Tables 1 and 2).

**Table 1:** Mean levels of systolic blood pressure (SBP) and diastolic blood pressure (DBP), and mean number of anti-hypertensive drugs in the initial visit (V1) and final visit (Vf). Goiânia - GO

	V1 (n=71)	Vf (n=71)	p
SBP (mm Hg)	157.3 ± 21.5	142.1 ± 20.9	<0.001
DBP (mm Hg)	95.1 ± 13.9	77.8 ± 10.8	<0.001
Number of drugs	1.49 ± 0.9	2.85 ± 1.2	<0.001

Student t test of related samples; significant: p<0.05; values expressed as means ± standard deviations.

**Table 2:** Distribution of the classes of anti-hypertensive drugs in the initial visit (V1) and final visit (Vf). Goiânia – GO

	V1	Vf	p
Diuretic	53.5% (38)	60.6% (43)	0.511
ACEI	22.5% (16)	46.5% (33)	0.004
ARB	4.2% (3)	35.2% (25)	<0.001
CCB	18.3% (13)	67.6% (48)	<0.001
BB	16.9% (12)	16.9% (12)	-
Spironolactone	0	5.63% (4)	0.125
Others	29.6% (21)	16.9% (12)	0.078

McNemar test; significant: p<0.05; values expressed as percentage and absolute numbers; ACEI: angiotensin-converting-enzyme inhibitor; ARB: angiotensin receptor blocker; CCB: calcium-channel blocker; BB: beta-blocker.

Analyzing the pharmacological treatment and comparing the initial and final prescriptions, a significant increase in the use of both statins (1.4% x 52.1%; p<0.001) and acetylsalicylic acid (11.3% x 39.4%; p<0.001) was found.

Analysis of laboratory variables evidenced an improvement in TC and LDL, after the institution of multiprofessional treatment, but worsening of the glomerular filtration rate during follow-up (Table 3).

**Table 3:** Mean levels of laboratory variables in the initial visit (V1) and final visit (Vf). Goiânia – GO

	V1	Vf	p
TC (mg/dL)	217.9 ± 40.5	191 ± 37.3	<0.001
HDL (mg/dL)	47.7 ± 9.8	47.3 ± 11.5	0.772
LDL (mg/dL)	139.6 ± 30.9	119.0 ± 33.2	<0.001
Triglycerides (mg/dL)	135.04 ± 66.85	122.48 ± 50.7	0.101
Glycemia (mg/dL)	102.5 ± 46.9	103.82 ± 29.7	0.819
GFR (mL/min)	62.5 ± 25.7	45.4 ± 15.2	<0.001

Student t test of related samples; significant: p<0.05; values expressed as means ± standard deviations; TC: total cholesterol; HDL: HDL-cholesterol; LDL: LDL-cholesterol; GFR: glomerular filtration rate.

Regarding lifestyle habits, no change was observed in the prevalence of smoking (5.6% x 1.4%; p=0.250) and of sedentary lifestyle (14.1% x 8.5%; p=0.388), but a significant reduction in the prevalence of alcoholism was observed with multiprofessional treatment (11.3% x 1.4%; p=0.039).

The patients' BMI decreased during follow-up, from 27.01 kg/m^2^ to 25.6 kg/m^2^ (p=0.001).

Regarding the comorbidities studied, the number of diabetic patients increased (8.5% x 28.2%; p<0.001), as increased the number of dyslipidemic patients (66.2% x 74.6%; p=0.345), but with no statistical significance for dyslipidemia. Only two cardiovascular events occurred in the population studied during follow-up.

## Discussion

Several national and international studies^7-10,15^ have shown the superiority of BP control with the multiprofessional treatment as compared to the conventional treatment. That evidence, however, is not available for very elderly hypertensives. This study showed a significant increase in the BP control rate, with 83.1% of the very elderly hypertensives on multiprofessional treatment showing BP control by the end of follow-up. That exceeds the BP control rates in very elderly hypertensives reported in different clinical contexts. In that age group, North American epidemiological data have shown BP control rates of 30.4%, between 1988 and 1994, and of 53.1%, between 2005 and 2010.^16^

The multiprofessional treatment of very elderly hypertensives reduced SBP levels by 15 mm Hg and DBP levels by 17 mm Hg. That more marked reduction in DBP as compared to SBP has been shown in other studies with the very elderly.^17,18^ Similarly, the increase in the number of anti-hypertensive drugs used during follow-up in this study has also been reported in other follow-up analyses of elderly hypertensives.^19^

One marked feature of the pharmacological treatment observed in this study regards the therapeutic regimen optimization adopted during follow-up, characterized by the increased use of first-line drugs, such as ACEIs, ARBs and CCBs. This suggests the good quality of care provided, with alignment of the pharmacological treatment with the recommendations of current guidelines.^11,12^

Another relevant aspect of the pharmacological treatment was the increasing use of statins and acetylsalicylic acid to our patients during follow-up. This indicates the excellence of the treatment conducted by the multidisciplinary team, abiding by guidelines on cardiovascular disease prevention.^20,21^

Regarding the laboratory findings, there was a significant reduction in TC and LDL levels, despite the population's aging. This can be explained by the increase in the use of statins. However, despite the significant BP reduction, the glomerular filtration rate worsened during the 15-year follow-up. This can also be explained by the population's aging, because renal function loss is known to be progressive from the age of 40 years onward.^22^

The lifestyle change of the very elderly patients studied was small. This is expected for an octogenarian population, because age is one of the greatest limiting factors of lifestyle changes.^23,24^ Nevertheless, there was a significant reduction in alcoholism in the group assessed.

Regarding nutrition, aging is associated with a decline in undernutrition and an expressive increase in obesity prevalence.^25,26^ Obesity is not simply weight increase, but excessive body fat. Aging is associated with increased fatty mass and changes in its distribution pattern, with a 20% to 30% increase in total body fat (2% to 5%/decade, after the age of 40 years).^27,28^ The BMI reduction observed in our sample has been reported in follow-up studies of elderly patients;^29^ however, taking only BMI into consideration is a superficial way to assess the nutritional status of the elderly.

Of the comorbidities considered in this study, a significant increase in new cases of diabetes was demonstrated and reproduces the findings of long-term follow-up studies of hypertensive patients.^29,30^ This is in accordance with the degenerative character of diabetes, widely demonstrated in observational studies, even in non-elderly.^31,32^

It is worth noting the reduced number of events in the group studied, which should be further investigated. Even the patients being very elderly and the follow-up time prolonged, only two cardiovascular events occurred. This might indicate that the multiprofessional treatment is capable of reducing cardiovascular outcomes in the very elderly.

One limitation of this study is its retrospective character. However, the fact that data collection was performed in a structured service since its conception for the generation of scientific knowledge reduces that limitation. The structure of the medical records is objective, and the completion of its mandatory fields is exhaustively trained. This ensures the ability of generating reliable data, although not prospectively.

In addition, the follow-up time was not homogeneous in this sample. Therefore, a minimum number of three consultations in our service was an inclusion criterion, ensuring not only the patient's minimum commitment to the service, but care provision by at least two professionals of different health areas within those three consultations. In addition, a more careful analysis of that follow-up time shows that most patients (85.9%) underwent the multiprofessional management for at least 5 years.

Another limitation was the lack of a control group. There was no comparison with a similar group, because all our patients undergo the same multidisciplinary treatment. This study compared the initial visit at the multidisciplinary service, when the patients were not on a multidisciplinary anti-hypertensive treatment, with the final visit, when they were already on the multiprofessional treatment. The use of a control group would be ideal for this study; however, the way we compared indirectly two treatment patterns in a little studied group of difficult follow-up should be highlighted. This study generates hypotheses, because the multiprofessional management of very elderly hypertensives has not yet been assessed with any methodology. In addition, the extremely positive results found will encourage further studies on that type of treatment, as well as its more comprehensive use.

The perspectives of investigating multiprofessional interventions in very elderly hypertensives are innumerous and extremely promising. Our data suggest that, similarly to other subgroups of hypertensive patients, the very elderly do benefit from a strategy of multifaceted treatment, which provides a more comprehensive and effective therapy.

## Conclusions

The multiprofessional intervention in very elderly hypertensives reduced BP and increased its control rate, with optimization of the pharmacological treatment instituted.
